# High-resolution frequency tuning but not temporal coding in the human cochlea

**DOI:** 10.1371/journal.pbio.2005164

**Published:** 2018-10-15

**Authors:** Eric Verschooten, Christian Desloovere, Philip X. Joris

**Affiliations:** 1 Laboratory of Auditory Neurophysiology, KU Leuven, Leuven, Belgium; 2 Department of Otorhinolaryngology, Head and Neck Surgery, KU Leuven, Leuven, Belgium; McGill University, Canada

## Abstract

Frequency tuning and phase-locking are two fundamental properties generated in the cochlea, enabling but also limiting the coding of sounds by the auditory nerve (AN). In humans, these limits are unknown, but high resolution has been postulated for both properties. Electrophysiological recordings from the AN of normal-hearing volunteers indicate that human frequency tuning, but not phase-locking, exceeds the resolution observed in animal models.

## Author summary

The coding of sounds by the cochlea depends on two primary properties: frequency selectivity, which refers to the ability to separate sounds into their different frequency components, and phase-locking, which refers to the neural coding of the temporal waveform of these components. These properties have been well characterized in animals using neurophysiological recordings from single neurons of the auditory nerve (AN), but this approach is not feasible in humans. As a result, there is considerable controversy as to how these two properties may differ between humans and the small animals typically used in neurophysiological studies. It has been proposed that humans excel both in frequency selectivity and in the range of frequencies over which they have phase-locking. We developed a technique to quantify these properties using mass potentials from the AN, recorded via the middle ear in human volunteers with normal hearing. We find that humans have unusually sharp frequency tuning but that the upper frequency limit of phase-locking is at best similar to—and more likely lower than—that of the nonhuman animals conventionally used in experiments.

## Introduction

The cochlea decomposes sound into bands of frequencies and encodes the temporal waveform in these bands, generating frequency tuning and phase-locking in the auditory nerve (AN). The relative roles of these two processes in human perception have long been debated [1,2] and would be clarified by knowing their limits. For example, studies in animals show that the average firing rate of AN fibers codes the spectral envelope of human vowels, but this code is problematic at high sound intensities. In contrast, a code based on phase-locking of the sound’s waveform (its “fine-structure,” i.e., the fast fluctuations in instantaneous pressure) is adequate at all intensities but does not extend above a few kilohertz. The difficulties of both coding schemes in explaining human perceptual ranges may reflect physiological differences in resolution between animal models and humans, in whom single fibers cannot be studied. The problem of rate coding at high sound levels may reflect broader spectral filtering in animal models [3]. This is supported by indirect estimates that report exceptionally sharp frequency tuning in humans, using behavioral estimates or otoacoustic emissions in subjects with normal hearing [4,5] and mass potentials in patients [6,7]. However, this conclusion is disputed [8–10]. The upper frequency limit of phase-locking is species dependent [11] but is unknown in human. Some perceptual abilities suggest use of temporal cues at 10 kHz or higher [12–16], but binaural sensitivity implies an upper limit barely above 1 kHz [17,18]. In summary, the present evidence regarding the limits of frequency tuning and phase-locking is conflicting. Knowledge of these limits is also important to understand and treat human hearing impairment [12,13,19].

We modified a clinical electrophysiological method [6,7,20,21] to study the AN in normal-hearing humans and macaque monkeys. An electrode is inserted through the eardrum to record potentials from the cochlear bony capsule. Combining a closed acoustic system calibrated in situ, stable trans-tympanic electrode placement under visual control, and validated stimulus and analysis paradigms [22,23], we studied the AN over several hours. Frequency tuning was obtained using pure tones to probe the imprint of a spectrally manipulated preceding notched-noise masker on the compound action potential (CAP; the summed response of AN fibers at the onset of the probe tone). Neural phase-locking was assessed with a paradigm separating the nonmaskable cochlear microphonic (CM) generated by hair cells, from the AN neurophonic. We achieved our aim of measuring both frequency tuning and the limit of phase-locking in humans and macaque monkeys and found that humans are unusual in the sharpness of frequency tuning but do not excel in the upper frequency limit of phase-locking.

## Results

### Frequency tuning

**Fig 1:**
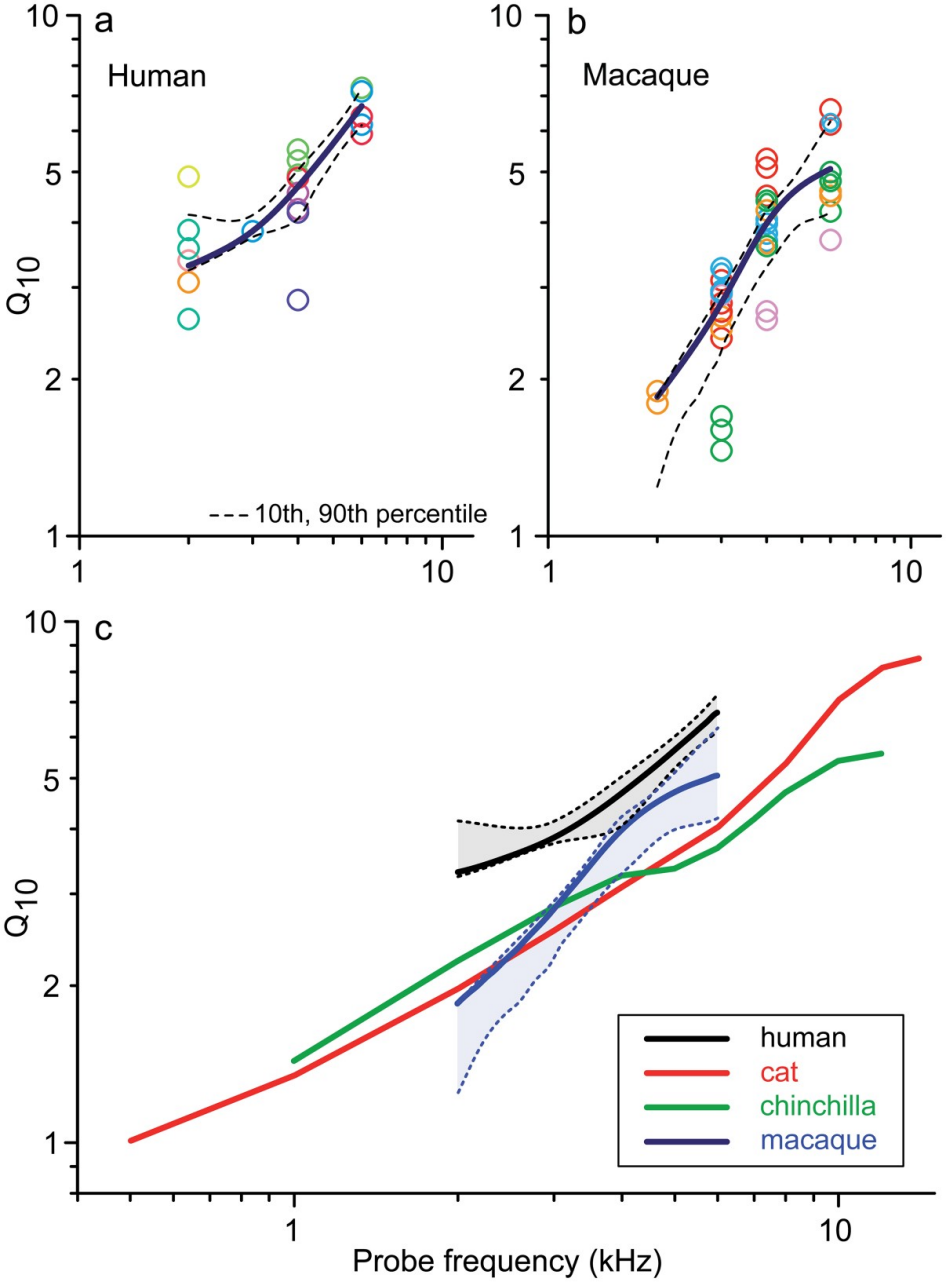
Sharpness of frequency tuning (Q_10_) increases with frequency and is higher in humans than in animal models tested. Values are CAP-Q_10_ results obtained with an NNFM paradigm. (a) Humans (*n* = 9); (b) macaque monkeys (*n* = 5). Different colors indicate different subjects; black lines are trendlines (Robust-LOESS, see Materials and methods); dashed lines indicate 10th and 90th percentiles of the trendlines obtained by resampling (bootstrapping, see Materials and methods). (c) Comparison of CAP-Q_10_ trendlines in human and monkey (from panels a and b) with cat and chinchilla [22]; shaded areas indicate the area between the 10th and 90th percentile of panels a and b. Underlying data provided in S1 Data. CAP, compound action potential; NNFM, notched-noise forward-masking; Q_10_, 10 dB quality factor.

**Fig 2:**
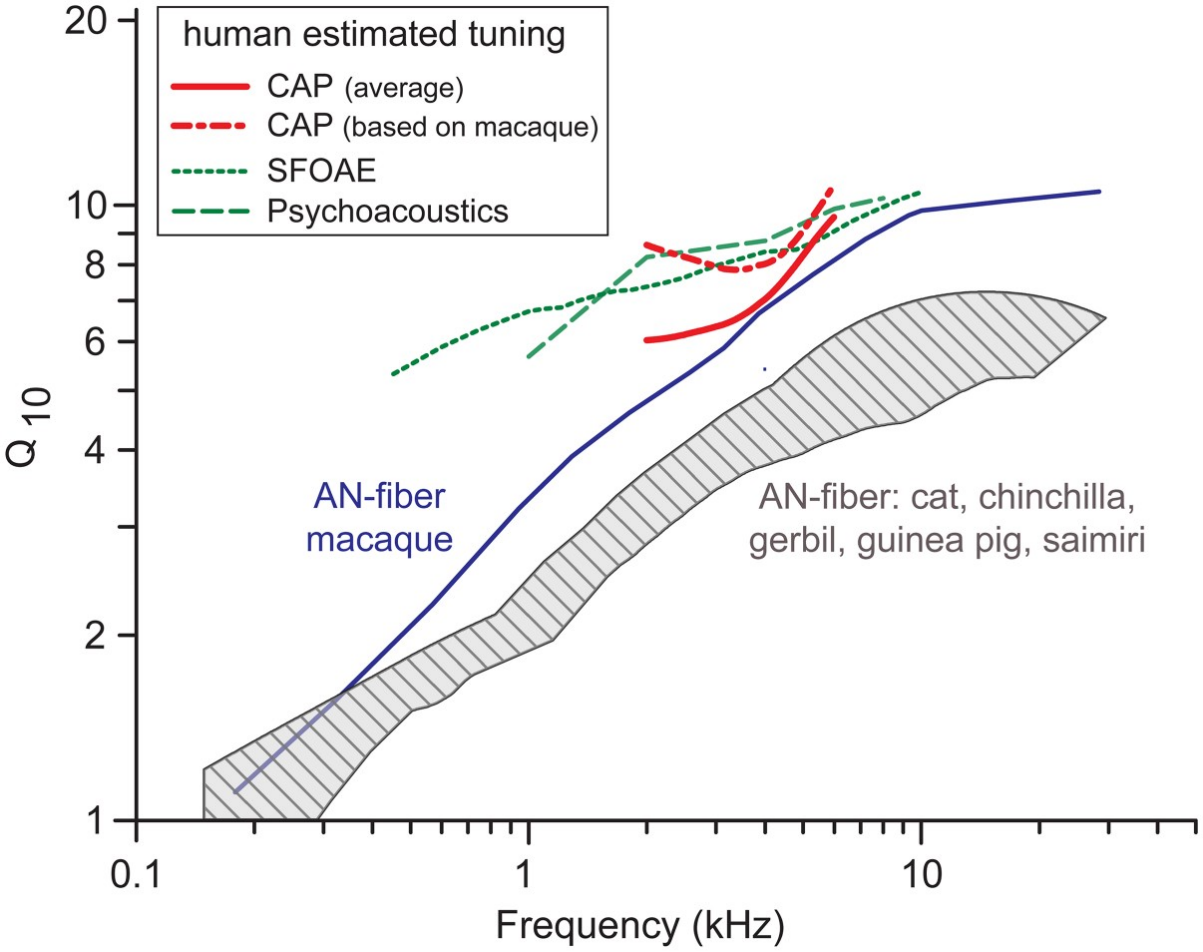
Estimates of frequency tuning of AN single fibers in humans. Estimates of sharpness of frequency tuning in single AN fibers of human (red) compared with single-fiber tuning of macaque [24] (blue) and other animal models (hatched area), and with other measures of human frequency tuning (green: dotted line, SFOAE [5]; dashed line, psychophysics [4]; both are converted from QERB with conversion factor 0.52). Red solid line: human estimate using conversion curve averaged across 3 species; red dashed line: estimate solely based on conversion curve of macaque. The hatched area outlines data for cat (archival data from our laboratory) and 4 other species [8]. Data provided in S1 Data. AN, auditory nerve; CAP, compound action potential; QERB, quality factor of Equivalent Rectangular Bandwidth; SFOAE, stimulus-frequency otoacoustic emissions.

The sharpness of frequency tuning obtained with the notched-noise forward-masking (NNFM) paradigm is shown in Fig 1 as a quality factor (Q_10_). Both human (Fig 1a) and monkey (Fig 1b) show a monotonic increase with probe frequency, consistent with other species [22,24] and with the vast literature on single AN fibers, but Q_10_ values in humans are significantly higher than in other species (Fig 1c) when compared over the same frequency range (average factors: approximately 1.6 times cat and chinchilla and 1.3 times monkey). CAP-based Q_10_ values differ from values in single AN fibers [22], which are the ultimate reference but cannot be studied in humans. However, availability of the 2 sets of data (CAP and single unit) in animals allows calculation of conversion functions based on the ratio between single-fiber Q_10_ and CAP-Q_10_ as a function of frequency. Applying the average of the conversion functions for cat, chinchilla, and macaque (S5a–S5c Fig) to measured human CAP-Q_10_ values, we predict human single-fiber Q_10_ to be slightly above those for macaque monkey [24] (red versus blue solid line, Fig 2).

Using only the monkey conversion function, which is arguably the most relevant, the predicted human single-fiber trend is even higher (red dotted-dashed line, Fig 2) and is remarkably consistent with assessments using nonelectrophysiological techniques [4,5] (Fig 2, green lines). Both predicted trendlines are higher than Q_10_ values reported for smaller, nonprimate animal models (Fig 2, red lines versus shaded area).

### Neural phase-locking

**Fig 3:**
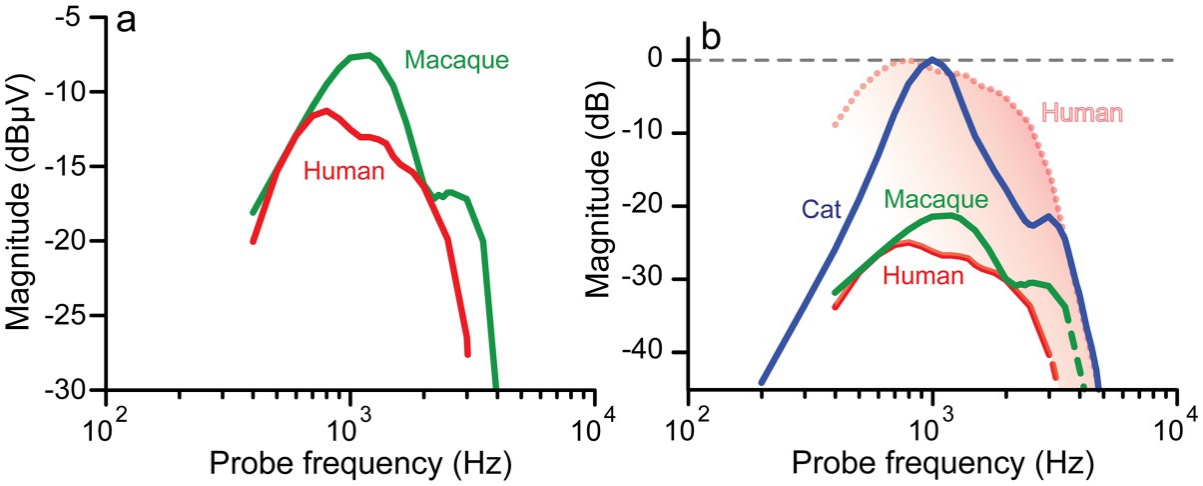
Phase-locking in humans extracted from neurophonic data. (a) Averaged trendlines for human and macaque. (b) Same data anchored to and overlaid with trendline for cat [23]. Dotted line: human trendline normalized to maximum amplitude in cat. Data provided in S1 Data.

Phase-locking in human and monkey showed the band-pass characteristic previously observed in similar measurements of the cat [23]. Maximal absolute amplitudes are only 4 dB smaller in human than in macaque; both are smaller than in cat [23]. The center frequency at the maximal absolute amplitude and the steep upper-frequency slope were lower in human (at approximately 0.7 and 3 kHz) than in monkey (at approximately 1 and 4 kHz) (Fig 3a). At face value, the data suggest that the upper phase-locking limit is lowest in human. However, several factors affect the absolute amplitude of the measured signal so that there is an unknown vertical offset between data for different species. For example, the recordings in cat were taken with a ball electrode on the round window, while in humans and monkeys, a needle was placed on the cochlear bony capsule, which provided a much smaller signal. In Fig 3b, single-fiber data are used to anchor cat and monkey data (S9 Fig) while keeping the relative position of monkey and human data. The frequencies (kilohertz) at which the trendlines cross the abscissa are 4.7 (cat), 4.1 (monkey), and 3.3 (human). Alternatively, the data for humans can be normalized to the maximum observed in cat (Fig 3b, dotted line)—even then, there is no suggestion of a higher limit of phase-locking in human than in cat.

## Discussion

We obtained the first electrophysiological recordings, to our knowledge, of cochlear potentials, which address both frequency tuning and temporal coding in humans with normal hearing. It is generally agreed that both processes have critical roles in human auditory perception, but there is considerable controversy regarding their relative roles, as well as regarding their resolution when compared to animal models. Impaired frequency selectivity and phase-locking have both been proposed as main causes for human hearing impairment [12,13,19].

The importance and presence of frequency tuning in humans is not under discussion, but 2 early studies that concluded that human frequency tuning is exceptionally sharp [4–6] were subsequently contradicted by different data or analyses [8–10]. Our data, which are electrophysiological and fundamentally different in nature from the previous estimates based on cochlear emissions and behavior [4,5], are strikingly in line with these earlier estimates (Fig 2).

An earlier study using a recording technique similar to ours [6] shows 2 CAP-Q_10_ values obtained with a tonal forward-masking paradigm at 8 kHz from 2 subjects with nominally normal hearing: these values (6.2 and 8.2) are reasonably in line with (somewhat lower than) the trend of our measurements extending to 6 kHz (Fig 1). From these measurements, the authors propose that human frequency tuning is sharper than in guinea pig and chinchilla but is rather similar to that in cat, which is not what we find when the same CAP-Q_10_ measurements are obtained in these different species (Figs 1 and 2 and [22]). Recent behavioral data suggest that frequency tuning in monkeys is not as sharp as in humans [25,26], consistent with our physiological data (Fig 1).

The situation is somewhat different for coding of fine-structure in humans, for which the discussion has been entirely based on behavioral research, and no attempt has been made to obtain direct measurements in humans. It is undisputed that coding of sound fine-structure is a prerequisite for binaural temporal sensitivity at low frequencies, with an abrupt upper limit at approximately 1.3 kHz [27,28]. Such coding has been proposed to be important for other auditory attributes as well, at frequencies as high as 10 kHz or more [12–16], but this is debated [17,29]. Using a validated technique [23] to extract neural phase-locking from the potentials measured near the cochlea, we find a reduced upper limit of phase-locking in monkey relative to cat, and in human relative to monkey. The consistency of these limits with those obtained in studies of single AN fibers (for cat and monkey) argue that phase-locking in human is limited to lower—rather than higher—frequencies than in commonly used laboratory species.

Our findings suggest a reappraisal of the fundamental debate that has been ongoing in hearing science for more than a century, regarding the importance of temporal versus “place” coding. This debate has taken various forms but in the past decades has centered on different codes available in the AN. As one example, in studies of the coding of human speech sounds by the firing rate of single AN fibers in small laboratory animals, frequency selectivity and dynamic range were not sufficient to code spectral features over the behaviorally relevant range [2,3,30]. On the other hand, phase-locking can account for the extremely wide perceptual intensity range but is limited in the upper frequency to which such coding is present, and it remains unclear how that temporal information can be extracted by the central nervous system. Our results suggest that the limits faced by models of “place coding” are less severe, and those by models of “temporal coding” more severe, than was thought based on data obtained from the small animal species used in neurophysiological experiments. For place coding they are less severe because the place map in the human cochlea is expected to be more fine-grained than in the experimental species studied [1,3]. For temporal coding the phase-locking limit is more severe because the fine-structure of sounds will not be coded up to the high frequencies at which it is, e.g., in the cat (about 5 kHz) [31].

Of course, humans differ from the other species studied along many dimensions, and it is at this point unclear how unique sharp frequency tuning is among mammals. A common, simple reasoning is that a small number of octaves “packaged” into a long cochlea will result in sharper frequency tuning than a large number of octaves subserved by a short cochlea [3,6,32–34]. Implicitly, this reasoning assumes that frequency tuning is limited by an absolute distance on the basilar membrane, which is similar across species. Measurements of cochlear dimensions in skulls of fossil and extant mammals [35] suggest that the cochlea of modern humans is “hypertrophied” relative to expectations on body size, so perhaps there is something special about the human use of hearing which drove sharper frequency selectivity. On the other hand, comparative studies suggest that larger animals have sharper tuning [32,33], so possibly human tuning is only remarkable in sharpness when compared to the (small) species studied experimentally. Independent of this issue, the sharper tuning observed in humans relative to species used in physiological studies indicates that inferences toward human perception have to be made cautiously, particularly when spectral versus temporal schemes are considered.

Data in animals were obtained under general anesthesia, whereas in humans only a local anesthetic was used: could our higher human Q-values be caused by this methodological difference? Effects of activation of the middle ear reflex can be excluded because we were careful not to evoke this reflex. First, the reflex intensity threshold was measured in all subjects (using tympanometry, see Materials and methods). Second, activation of the middle ear reflex can be monitored during the recordings because muscle action potentials strongly contaminate the neural recordings. We are also quite confident that efferents of the medial olivo-cochlear (MOC) system did not contribute to the sharper tuning observed in humans because we explicitly looked for efferent effects (on CAP or neurophonic amplitude) in separate experiments and found them to be very small (a few decibels), and biased towards low frequencies [36]. The MOC reflex is functional in both anesthetized and decerebrated cats [37], and no difference in CAP tuning was found in awake versus anesthetized guinea pigs [6]. Finally, the expectation from animal work is that efferent effects would cause a reduction in sharpness of tuning, so that if only present in awake humans, they would have tended to make the difference between Q-values in humans and anesthetized animals smaller rather than larger.

There are marked difference in length of the ANs of the species studied (factor of approximately 4 between cat and human): could the differences that we measured in the upper-frequency limit of phase-locking reflect spatial integration of the propagating action potentials along the AN? Although this issue can only be directly addressed by recordings from individual nerve fibers, there are several arguments against such spatial integration. It would cause significant low-pass filtering of the CAP in human, while we find that the initial negative waveform in humans and cats is very similar (e.g., S2 Fig). Also, studies of mass potentials [38–40] and of unit contributions of single nerve fibers [41,42] suggest that these potentials reflect a potential difference generated over a restricted segment of the AN rather than spatially distributed generators.

In summary, we provide electrophysiological evidence that our species excels in sharpness of frequency tuning but not in temporal coding of fine-structure. This dual result calls for a reappraisal of coding schemes based on average firing rate, e.g., for the coding of pitch and speech. The plausibility of such schemes relative to temporal schemes may have been unduly dismissed based on the more limited resolution of place-rate coding in experimental animals on the one hand, and unrealistic assumptions regarding the extent of temporal coding in humans on the other hand.

## Materials and methods

### Subjects

Human experiments were carried out in accordance with the recommendations of good clinical practice (ICH/GCP) and were approved by the Medical Ethics Committee of the University of Leuven. All subjects gave written informed consent in accordance with the Declaration of Helsinki. Human volunteers were recruited on campus with advertisements. A total of 19 subjects (15 female, 4 male) participated in the experiments. Participants were between 20 and 35 years old and received a financial compensation. Frequency tuning data were based on recordings from 9 subjects; for neural phase-locking, data of 7 subjects were used. In 4 subjects, both types of data were recorded. In the remaining 7 subjects, no useable data were obtained, for various reasons. In 3 subjects, the signal-to-noise ratio (SNR) was too poor (in 1 restless person due to excessive muscle artifacts and in the others for unknown reasons). In 2 subjects, the needle could not be placed at the desired location because of a narrow and heavily curved ear canal. In 2 subjects, no measurements could be started due to practical issues.

Animal procedures were approved by the Animal Ethics Committee of the University of Leuven. Recordings in Monkey were obtained from 1 ear in 4 rhesus monkeys (*Macaca mulatta*), which were also involved in chronic visual experiments (an 8.9-kg adult male, a 4.8-kg juvenile male, and 2 juvenile females of 6.3 kg and 4.7 kg; ages were between 4 and 7 years). Prior to the experiments, dissections on formaline-preserved temporal bones were performed to study the best trajectory and practice the placement of the needle electrode.

### Screening of subjects

The day before—or morning of—the session, the hearing of the volunteers was screened, including an inquiry for hearing problems, a pure tone audiogram (thresholds <20 dB nHL, 125 Hz–8 kHz), tympanometry to assess middle ear function, and an otoscopic examination by an otolaryngologist. Subjects were requested to avoid exposure to loud sounds in the days preceding the experimental session. For monkey, the tympanic membrane was otoscopically checked after induction of the anesthesia.

### Anesthetics

Human subjects were unsedated during the experiment. Before insertion of the needle electrode, the tympanic membrane and ear canal were locally anesthetized with Bonain’s solution (equal volumes of cocaine hydrochloride, phenol, and menthol; aspirated after 30 minutes). Subjects usually had a short-lasting and vague sensation of touch during insertion of the electrode, which quickly disappeared.

Recording in monkey was similar to that in human, with the main difference being the presence of general anesthesia. Induction was done with a mixture of ketamine (3 mg/kg) and medetomidine (intramuscular, 0.050 mg/kg). The same mixture was administered intravenously for maintenance through a venous cannula inserted for administration of lactated Ringer’s solution. The duration of the total experimental session, including the placement of the electrode, was between 4 and 6 hours. After the experiments, atipamezole (intramuscular, 0.2 mg/kg) was administered to reverse the sedative effect of medetomidine; after awakening of the animal, it was observed until it was freely moving about.

### Experimental apparatus

To minimize electrical and acoustical interference, all experiments were conducted in a double-walled soundproofed and faradized booth (Industrial Acoustics Company, Niederkrüchten, Germany). Before the experiment, human subjects chose a comfortable supine position on a bed and were asked to remain still during the trans-tympanic insertion of the needle electrode (TECA; sterile monopolar disposable, 75 mm × 26 G, 902-DMG75-TP) and the actual recordings. While in the sound booth, subjects and experimentalists were grounded to the booth via an antistatic wrist strap.

Anesthetized monkeys were positioned on a heating pad with their heads restricted in a stereotactic frame and turned for ease of needle insertion. Core body temperature was maintained using a feedback-controlled homeothermic system (Harvard Apparatus, Model 50–7129). Eyes were coated with a thin layer of ophthalmic ointment (Pfizer, Terramycine) to prevent desiccation.

For every human subject, a custom silicone earmold (Dentsply, Aquasil Ultra XLV regular) was made for acoustical reproducibility throughout the procedure and to preserve low frequency performance of the earphone speaker (Etymotic, ER-2 or ER-1). The earmold contained 2 casted openings for different manipulations, such as needle insertion, visualization, acoustic stimulation, and calibration. During most actions (e.g., placing of the needle electrode through one of the earmold’s openings), the ear canal and tympanic membrane were visualized by a rigid endoscope with camera (R. WOLF, 8654.402 25 degree PANOVIEW; ILO electronic GmbH, XE50-eco X-TFT-USB) through the other available opening of the earmold. In order to maintain the position of the needle electrode relative to the unrestricted head in human, a custom frame—consisting of a ring that was centered above the external ear and fastened around the subject’s head with Velcro straps—was used. On this ring, a needle holder allowed stable support of the needle electrode under slight tension in order to maintain good electrical contact.

In monkey, the recording needle was secured by a mechanical micro-manipulator mounted on the stereotactic frame. The placement of the needle electrode was performed while visualizing the ear canal and tympanic membrane with a surgical microscope (ZEISS, OPMI pico). Earmolds were made in situ, after placement of the needle electrode, with ear impression compound (Microsonic).

### In situ calibration

The calibration of the acoustic system (ear canal and earmold) was performed in situ with a closed-loop system using a tube earphone speaker (Etymotic Research, ER1 or ER2) and a microphone (Etymotic Research, ER-7C) with a silicon probe close to the tympanic membrane. In humans, the calibration was done before placement of the needle electrode. In monkeys, calibration was performed after placement of the needle electrode with the silicon probe tube embedded in the earmold. Sound was delivered through one of the openings of the earmold via a plastic T-piece, which allowed access for the endoscope. During calibration and recording, all openings were sealed airtight except for a tiny opening in the plastic T-piece that prevented static pressure build-up.

### Trans-tympanic electrode placement

A trans-tympanic procedure was developed, extensively tested, and practiced on more than 20 fresh human cadavers in the university hospital. The sterile needle electrode was inserted by an ENT surgeon through one of the openings of the earmold that contained a short sterile plastic tube (length <1 cm; diameter 2 mm). The needle electrode was placed, trans-tympanically (3rd quadrant), on the cochlear promontory or in the niche of the round window. The experimental session was terminated within 4 hours or when the subject expressed the desire to stop. The needle electrode was then pulled back, and the earmold was removed. The session was concluded with an otomicroscopic examination. In no cases was there an eardrum perforation larger than expected from the needle’s diameter (0.46 mm). Subjects were requested to keep the ear dry for 10 days following the recording session. An otolaryngologist was available during the weeks after the experiment to address any worries or for an additional checkup.

### Acoustical stimulation

Stimuli were generated with custom software and a digital sound system (Tucker-Davis Technologies, system 2, sample rate: 125 kHz/channel) consisting of electromagnetically shielded earphone speaker (Etymotic Research, ER-1 or ER-2), a headphone driver (HB7), a digitally controlled analog attenuator (PA5), and a digital-to-analog converter (PD1).

### Electrophysiological recordings

Acoustically evoked cochlear mass potentials were recorded using a low-noise differential preamplifier (Stanford Research Systems, SR560), as described in our previous publication [36]. The signal input was connected to the trans-tympanic needle electrode, the reference input was connected to an earlobe clamp coated with conductive gel, and the ground input was connected to a standard disposable surface electrode placed at the mastoid, also coated with conductive gel. All contacts were made on the side ipsilateral to the recording. The battery-operated preamplifier was galvanically isolated (A-M systems, Analog stimulus isolator Model 2200) from the mains-powered equipment. Before the signal was recorded (TDT, RX8, approximately 100 kHz/channel, maximum SNR 96 dB), stored, and analyzed (The Mathworks, Matlab), the signal was further amplified (DAGAN, BVC-700A) to a total gain of × 100 k and band-pass filtered (30 Hz–30 kHz; cut-off slopes 12 dB/octave). During the sessions, the most relevant signals were visualized on an oscilloscope (LeCroy, WaveSurfer 24Xs) and monitored with a loudspeaker outside the experimental booth.

### Data processing

Recordings were averaged off-line over multiple repetitions (between 128 and 1,024, depending on background noise level) to increase SNR. CAP responses were obtained by summing responses with alternating stimulus polarity and were additionally de-noised with a band-pass filter in range of the spectrum of the CAP. CAP amplitudes were measured between the first negative trough (N1) and first positive peak (P1), or if P1 was not clearly defined, between N1 and the second positive peak (P2); otherwise, they were measured between N1 and the positive maximum (S2 Fig).

### Experimental paradigm, frequency tuning

CAPs reflect activity of many AN fibers [41,43] but are not frequency selective. To assess frequency selectivity, we used a modified NNFM paradigm [4,44] to extract masking tuning curves (MTCs). Briefly, this involves measuring the CAP to a probe signal that is a short pure tone, fixed in level and frequency. The probe level is fixed at the SPL that results in a SNR of 18 dB, when the probe is given by itself. The probe tone is then preceded by a noise (forward) masker, which results in a reduction of the CAP to the tone. First, a broadband noise masker is used whose SPL is adjusted so that that a CAP suppression of 33% is obtained. This masker is then increased 10 dB in level (causing more masking); a spectral notch is introduced centered at the frequency of the probe tone, and the notch width is then varied to search for the width restoring a CAP suppression of 33%.

#### Stimulus

A schematic representation of the stimulus in the time (a) and frequency domain (b) are shown in S1 Fig. A notched-noise forward masker (t_m_, approximately 150 ms) is followed by a brief pause (t_mp_, 10 ms) and a short tonal probe (t_p_, 10 ms) with fixed level. Probe and masker are gated with 5 ms raised-cosine ramps to reduce spectral splatter at ON- and OFF-switching of the stimuli. To cancel the CM (non-neural receptor potential), this stimulus sequence is repeated but with an inversion of the polarity of the probe tone. The silent interval (t_s_) between these 2 presentations (shown as blue and red) was 10 ms. The CM follows the polarity of the stimulus so that it is almost completely removed by averaging the responses to stimuli with alternating polarity. In contrast, the CAP is mainly from neural origin and is largely preserved after averaging. S1 Fig1b depicts the idealized frequency spectrum of the stimulus. Spectrally, 2 noise bands with equal bandwidth (f_p_/4) straddle the fixed probe frequency (f_p_) so as to create a notch symmetrically spaced around f_p_. The masker notch width and level are the main experimental variables that determine the extent of suppression of the CAP response.

#### Experimental procedure

The experimental procedure was as follows:

1. Fixed probe level: First, a suitable probe level (no masker) was sought, yielding a CAP waveform with a minimum SNR of 18 dB (S2 Fig). We refer to a probe tone at this level as the “predefined probe tone.”
2. Masker reference level: The predefined probe tone was delivered and a (no-notch) broadband masker was presented at several levels (S3 Fig): for each masker level, the CAP amplitude was measured and a curve (“masking curve”) was fit through these data points (S3 Fig). From this curve, the “masker reference level” was extracted, which is the masker level that suppresses the CAP response at the predefined masking criterion of 33% (dashed line, S3 Fig).
3. Q_10_: The predefined probe tone was presented together with a masker at a level 10 dB above the masker reference level (the Q_10_ level). When the masker is broadband, it will obviously generate stronger masking than 33%. A notch was now introduced in the broadband masker, and the masker notch width was varied to determine the width that resulted in the same amount of masking (target: 33%) as the (no-notch) broadband noise masker at the masker reference level (i.e., 10 dB lower in level). In practice, to save time, a range of preselected notch widths was delivered, and the notch width generating 33% of masking was obtained by interpolation of the responses to these different notch widths (see S4 Fig). The different notch width conditions, including that of the previously obtained masker reference level (no-notch masker condition, 10 dB below that of the notch-noise conditions), were typically presented as a single stimulus assembly, which was repeated to minimize variability between different conditions. The sharpness of tuning, expressed as a Q_10_, is the probe frequency divided by the obtained notch width (S4 Fig). The average measurement time needed for determination of 1 Q value was minimally 1 hour.

#### Relation between Q_10_ and stimulus level

CAPs measured at the bony capsule of the cochlea in humans and monkeys were smaller and noisier than those measured at the round window in cat and chinchilla. To comply with the predetermined SNR (18 dB), stimulus levels in human, relative to cat and chinchilla, needed to be approximately 30 dB higher for both the masker (human: 50–70 dB SPL; monkey 40–65 dB SPL) and the probe (human: 55–75 dB SPL; monkey 75–80 dB SPL). It is well known that cochlear frequency selectivity decreases with level. This has been extensively documented for iso-input measurements [45]. Modeling studies suggest that, for iso-response measurements, as used here, Q-values can actually increase with level [46] so that we need to consider the possibility that higher Q values in human are due to the higher stimulus levels used. However, in the NNFM CAP study in animals [22], such an increase in sharpness with probe level was not observed. On the contrary, for probe levels below approximately 50 dB SPL, there was almost no dependence of Q_10_ on probe level, and above this level, the Q_10_ was negatively correlated with probe level [22]. Consequently, if higher stimulus levels would have affected our Q estimates, they would give underestimates rather than overestimates.

#### Conversion functions

To make a prediction of human single AN fiber Q_10_ values based on the measured CAP Q_10_ values, we made use of conversion functions. These functions, based on data collected on 3 animal models, provide the ratio between single-fiber Q_10_ and CAP Q_10_ trendlines as a function of frequency (S5 Fig). The purple dashed-dotted lines are the conversion functions (ratios) between the AN fiber Q-values (green dashed lines) and CAP Q-values (red lines) for the 3 species, over the frequency range for which we measured CAP data in human (2 to 6 kHz). (a) The conversion function for cat is obtained with AN fiber data (dashed green) and Q_10_ data (red solid) from [22]. (b) The conversion function for chinchilla is obtained with AN fiber data from [47] and Q_10_ data from [22]. (c) The conversion function for macaque monkey is obtained with AN fiber data from [24] and Q_10_ data from Fig 1b.

### Experimental paradigm neural phase-locking

We assess neural phase-locking using a phase-locked neural component in the electrical mass potential recorded in the middle ear. Previously, we developed a method based on forward masking to disentangle the neural phase-locked component from that of the receptors (CM). We demonstrated the validity of this method in cat in two respects: that it isolated neural components and that it yielded an upper-frequency limit of neural phase-locking close to that reported in single AN fibers [23]. In human and monkey, the same stimulus paradigm was used as previously developed in cat [48]. Some parameters were adjusted to optimize measurement time. Briefly, the neural signal is disambiguated from the CM by comparing the response to a tonal probe with the response to the same probe but preceded by a masker. To then extract only the neural phase-locked component and discard the CAP, the responses to 2 opposite stimulus polarities are subtracted from each other.

#### Stimulus

A schematic representation of the stimulus with average response (green waveforms) used for neural phase-locking is illustrated in S6 Fig. The upper (red, S6 Fig, panel a) and lower (blue, S6 Fig, panel c) half stimulus representations are identical except for being inverted in polarity, and each consists of 3 segments. The first segment x contains only a probe, the second segment y contains a masker followed by a probe, and the last segment z contains only the masker. The probes (50 ms) and maskers (83.71 ms) were pure tones at the same frequency (f_P_). The masker-probe interval was 1 ms, and the interval between different stimuli was at least 10 ms. Segment z had a stimulus-free period of approximately 80 ms, which was required for the recovery of masking and was also used for the determination of the noise floor in the individual results. To reduce spectral splatter, the probe and masker were gated with a 1 ms raised-cosine. The parameters that were modified in order to optimize measurement time were the probe length (50 ms), probe-masker interval (10 ms), number of averages (n ≥ 200) and stimulus levels (probe level: 65, 70, or 75 dB SPL versus 50 or 55 dB SPL in cat; the masker level was always 10 dB above the probe level).

#### Analysis of responses, neural phase-locking

The analysis performed on the responses of human and monkey to obtain a measure for neural phase-locking was the same as in our previous study in cat [48], in which the responses to different segments (S6 Fig) were combined to obtain the required signals (S7 Fig). The first pair of responses (S7 Fig, panel A, trance a) is the basic alternated probe response (magenta: positive polarity [P]; cyan: negative polarity [N]) from the stimuli in segment x (S6 Fig). This response contains both neural (CAP, neurophonic) and receptor (CM) potentials. To extract the neurophonic, the other components (CM and CAP) have to be removed. Unfortunately, due to the tight relationship between the neurophonic and CM, it is impossible to remove or reduce the CM without also removing the neurophonic. However, the opposite is possible: the neural component (neurophonic and CAP) can be suppressed by using neural adaptation, a neural property that is not present for the CM. By preceding the probe with a sufficiently stronger signal (the masker), the neural response of the probe can be temporarily suppressed. The result of this is shown by the second pair of responses (S7 Fig, panel A, trace b) and is called the “masked response.” This pair is corrected for the masker’s trailing off-set response (S6 Fig, filled arrows) by subtracting this off-set response in segment z (S6 Fig, open arrows) from segment y. From this pair of corrected responses, a new pair is derived by subtracting the masked response (S7 Fig, panel A, trace b) from the probe response (S7 Fig, panel A, trace a): this we call the “adapted component” (S7 Fig, panel A, trace c). In a previous study [48], it was shown that the adapted component is neural in origin.

In this study, we are mainly interested in the fundamental phase-locked component, but the adapted component contains also the CAP and other harmonics. We canceled these unwanted neural components by subtraction of the halved pairs of responses ([P − N]/2) (S7 Fig, panel A); the result is shown in S7 Fig (panel B). The resulting response is stimulus polarity dependent and is dominated by the fundamental component. To quantify the decaying neural phase-locked signal (S7 Fig, panel B, trace c), we used the same method and settings as in our previous study [48]. The method obtains the maximum of the time course of the”instantaneous” magnitudes of only the fundamental component using a Gabor transform. The Gabor transform is a special case of a short-time Fourier transform (STFT; MATLAB, spectrogram) with a Gaussian time window. In this study, the Gaussian window was truncated at α = 2.5 and had a fixed defined window length of 6 cycles of the probe frequency. The STFT window was moved in steps of 100 μs with an overlap between 75% and 98%. The size of the FFT was chosen such that the frequency spacing between the spectral components was fixed to 2.5% of the desired frequency. For every time step, the center frequency (maximum power) was searched within a spectral range of ±10% around the desired frequency (here, 800 Hz). The magnitude as a function of time was obtained as the magnitude corresponding to the power calculated as the sum of the power of the spectral components within a range of ±20% on the center frequency (= 98.9% spectral coverage of the desired magnitude).

#### Noise floor compensation, neural phase-locking

For the assessment of the upper frequency limit of phase-locking, the signal noise floor was quantified. It was calculated from the background noise in the response- and artifact-free part of segment Z (e.g., S6 Fig, 320 to 366 ms). Over this time window, the background noise was stable over time (standard error of only a few tenths of a decibel) and was dominated by non-neural sources. This calculation was described in our previous publication [48] and involved, when possible, the response combinations and operations (e.g., subtraction, STFT, etc.) that were also used to quantify the responses. In case not all operations could be performed, the noise was compensated with a factor corresponding to these operations assuming uncorrelated noise. For example, the removal of the masker’s trailing off-set response (cf. arrows Fig 6) involves a subtraction which for the noise estimate was replaced by a multiplication with sqrt(2). Noise floors for the peak and STFT amplitude were calculated as the 99.75th percentiles (obtained with spline interpolation) of the noise distribution around the mean value of the processed noise plus the mean value itself. The reason for this seemingly insignificant operation was to reduce fluctuations in the baseline due to outliers in the noise.

Due to the low SNR in human (maximum 8 dB) and to a lesser extent in monkey (maximum 15 dB), at the highest frequencies, the neurophonic is overtaken by the noise floor (dotted lines and shading, S8 Fig). Because the spectrum of the noise floor is independent of the neurophonic and is known, the transfer functions of the measured neurophonic (dashed lines, S8 Fig) can be compensated for by the noise floor. The compensated transfer function of the neurophonic (solid lines, S8 Fig) is obtained by subtraction of the power of the noise floor from that of the neurophonic $({Neurophonic}_{Compensated}\;=\;\sqrt{{{Neurophonic}_{Measured}}^{2}-\;{Noise\;Floor}^{2}})\;$. As in our previous study [23], we defined the upper frequency limit of phase-locking (indicated by the horizontal dashes on the compensated trendlines) as the frequency 10 dB below the intersection with the noise floor (indicated by the empty circle and short dotted lines in S8 Fig).

### Statistical analysis

All data were processed and analyzed with custom MATLAB (The Mathworks) scripts. To improve the response’s SNR, the uncorrelated background noise was reduced by averaging the response of many repetitions (n > 127), and multiple Q-values were obtained at every measured frequency. Nevertheless, due to time constraints (2–4 hours) in awake humans and anesthetized monkey (4–6 hours), only a limited number of Q-values could be extracted in each subject. Therefore, the population data in human and monkey are not evenly distributed across frequencies. To cope with this unevenly distributed data and to minimize the influence of outliers, we obtained Robust-LOESS trend-values instead of mean-values. The LOESS is a nonparametric local regression function using weighted linear squares and a second-degree polynomial model. The Robust version, the RLOESS assigns lower weight to outliers in the regression. The weights are given by the bisquare function with 0 weight for deviations greater than 6 mean absolute deviations. In Fig1, the RLOESS trend-values were obtained using the MATLAB (The Matworks) *SMOOTH* function from the averaged Q-values within a subject. Moreover, the trendlines were obtained using a RLOESS function with a span of 0.85 and by interpolating the results with smoothing splines (FIT, MATLAB, option: “SmoothingSpline,” parameter: 0.999 approximate cubic spline). The 10th and 90th percentiles of the RLOESS trendline were estimated using bootstrapping [49] (*n* = 200), which is a random resampling method with replacement. The RLOESS function and bootstrapping were performed on the average of repetitions (same condition and experiment).

For the neurophonic (e.g., Fig 3a and S8 Fig) a similar approach was used to obtain the trendline. In this case, the LOESS function was used with span 0.55, and the resulting trend values were connected by straight lines. The bootstrap standard error for the frequency limit of phase-locking was approximately 270 Hz for monkey and approximately 450 Hz for human. Because the absolute amplitude of the neurophonic is not only dependent on neural factors (e.g., cochlea-dependent spatiotemporal summation, electrode contact impedance, etc.), it can differ between subjects. Therefore, the individual data were first normalized to their maximum value before the application of bootstrapping.

## Supporting information

S1 Fig Schematic representation of the stimulus for measurement of frequency tuning. (a) time domain, (b) frequency domain.
(AI) Click here for additional data file.

S2 Fig Two examples of human CAP response as a function of probe level (L_P_) for 2 different probe frequencies. Left panel for a probe frequency (f_P_) of 2 kHz; right panel for 6 kHz (different subject). The magnitude of the CAP is the maximum voltage as indicated in the figure (P1–N1). The response in blue (thicker line) in the left panel is an example of a response that meets our SNR criterion of 18 dB. Data are averaged but not de-noised. Data provided in S2 Data.
(AI) Click here for additional data file.

S3 Fig CAP masking as a function of masker level with indication of the masker reference level. (a) Example of masked CAP responses (not de-noised). The CAP is increasingly reduced or masked by increasing masker level. (b) Masking curves for different probe frequencies. The horizontal dashed line indicates the masking criterion. The vertical dashed lines and arrowheads indicate the corresponding masker reference levels for different subjects and different probe frequencies. The masker was a broadband noise. Data provided in S3 Data.
(AI) Click here for additional data file.

S4 Fig Masking as a function of normalized notch width. Masker levels were fixed at 10 dB above the masker reference level; their notch width was varied to bracket the level generating 33% masking (i.e., a reduction in CAP amplitude of 33%). The percent masking values are graphed as a function of normalized notch width (with respect to the probe frequency), and a trendline is fit through the masking values—this is the masking curve. The horizontal dashed line indicates the masking criterion (target: 33%). Vertical lines and arrows indicate the notch width giving the Q_10_, obtained by interpolation (crossing of masking criterion, 33%, by masking curve). Data are shown for 2 subjects. Probe frequency was 4 kHz. Data provided in S4 Data.
(AI) Click here for additional data file.

S5 Fig Conversion functions between sharpness of tuning (Q_10_) data obtained from single AN fibers or CAP. The purple dashed-dotted lines are the conversion functions (ratios) between the AN fiber data (green dashed lines) and CAP data (red lines) for the frequency range measured in human (2 to 6 kHz). (a) The conversion function for cat is obtained with AN fiber data (dashed green) and Q_10_ data (red solid) from [22]. (b) The conversion function for chinchilla is obtained with AN fiber data from [43] and Q_10_ data from [22]. (c) The conversion function for macaque monkey is obtained with AN fiber data from [24] and Q_10_ data from Fig 1b. Data provided in S5 Data.
(AI) Click here for additional data file.

S6 Fig Schematic representation of the stimulus for measurement of neural phase-locking, with averaged responses. Segment x contains the probe only, segment y contains the probe preceded by a masker, and segment z contains the masker only. The probe and masker shown are pure tones of 800 Hz; the stimuli in (c) (blue) are inverted in phase relative to those in (a) (red). The arrows indicate the offset response of the masker in segments y and z.
(AI) Click here for additional data file.

S7 Fig Example of averaged stimulus evoked responses recorded in human. (Aa) A pair of raw probe responses for opposite stimulus polarity. (Ab) Same as Aa, but with a preceding tonal masker (not shown). (Ac) Difference between signal Aa and Ab: the adapted component. (Ba–c) Difference of the response pairs shown in each of the traces of Aa, Ab, and Ac. Parameters: probe level = 75 dB SPL, masker level = 85 dB SPL, frequency = 800 Hz. The Aa traces contain all response components (receptor potential + neural). The initial part of the Ab traces contain only receptor potentials: the neural components are masked. The Ac traces contain only neural components: the components that were masked and therefore absent in Ab. These neural components contain both the CAP and the neurophonic: to eliminate the CAP, the response pairs to opposite polarities are subtracted from each other (Bc). Data provided in S6 Data.
(AI) Click here for additional data file.

S8 Fig Compensated neurophonic. The transfer functions of the neurophonic (dashed lines) for human (red) and monkey (green) are corrected (solid lines) for the influence of the noise floor (dotted lines with shadow). The 3 dB point is indicated by the empty circle, where the compensated signal is equal to the noise floor. Data provided in S7 Data.
(AI) Click here for additional data file.

S9 Fig Trend of maximum vector strength as a function of characteristic frequency for AN fibers of cat and macaque monkey. Near the upper limit of phase-locking (approximately 4 kHz), the trend in monkey is about 0.2 octave lower than in cat (indicated in the figure by the arrows). Data for cat [31] and macaque monkey are provided (S8 Data). A discussion of the low-pass shape of these functions versus the more band-pass shape of the neurophonic measurements (Fig 3) is provided by Verschooten et al. (2014). Data provided in S8 Data.
(AI) Click here for additional data file.

S1 Data Data of Figs 1, 2 and 3. (XLSX)

S2 Data Data of S2 Fig. (XLSX)

S3 Data Data of S3 Fig. (XLSX)

S4 Data Data of S4 Fig. (XLSX)

S5 Data Data of S5 Fig. (XLSX)

S6 Data Data of S7 Fig. (XLSX)

S7 Data Data of S8 Fig. (XLSX)

S8 Data Data of S9 Fig. (XLSX)

## Abbreviations

AN: auditory nerve
CAP: compound action potential
CM: cochlear microphonic
MOC: medial olivo-cochlear
MTC: masking tuning curve
NNFM: notched-noise forward-masking
Q_10_: 10 dB quality factor
SFOAE: stimulus-frequency otoacoustic emission
SNR: signal-to-noise ratio
